# Are Artificial Intelligence Models Listening Like Cardiologists? Bridging the Gap Between Artificial Intelligence and Clinical Reasoning in Heart-Sound Classification Using Explainable Artificial Intelligence

**DOI:** 10.3390/bioengineering12060558

**Published:** 2025-05-22

**Authors:** Sami Alrabie, Ahmed Barnawi

**Affiliations:** Faculty of Computing and Information Technology (FCIT), King Abdulaziz University, Jeddah 21589, Saudi Arabia; ambarnawi@kau.edu.sa

**Keywords:** heart-sound classification, attention mechanism, Grad-CAM, deep learning, explainable AI (XAI)

## Abstract

In recent years, deep learning has shown promise in automating heart-sound classification. Although this approach is fast, non-invasive, and cost-effective, its diagnostic accuracy still mainly depends on the clinician’s expertise, making it particularly challenging to detect rare or complex conditions. This study is motivated by two key concerns in the field of heart-sound classification. First, we observed that automatic heart-sound segmentation algorithms—commonly used for data augmentation—produce varying outcomes, raising concerns about the accuracy of both the segmentation process and the resulting classification performance. Second, we noticed inconsistent accuracy scores across different pretrained models, prompting the need for interpretable explanations to validate these results. We argue that without interpretability to support reported metrics, accuracy scores can be misleading because of ambiguity in how training data interact with pretrained models. Specifically, it remains unclear whether these models classify spectrogram images—generated from heart-sound signals—in a way that aligns with clinical reasoning, where experts focus on specific components of the heart cycle, such as S1, systole, S2, and diastole. To address this, we applied explainable AI (XAI) techniques with two primary objectives: (1) to assess whether the model truly focuses on clinically relevant features, thereby allowing classification results to be verified and trusted, and (2) to investigate whether incorporating attention mechanisms can improve both the performance and the model’s focus on meaningful segments of the signal. To the best of our knowledge, this is the first study conducted on a manually segmented dataset, which objectively evaluates the model’s behavior using XAI and explores performance enhancement by combining attention mechanisms with pretrained models. We employ the Grad-CAM method to visualize the model’s attention and gain insights into the decision-making process. The experimental results show that integrating multi-head attention significantly improves both the classification accuracy and interpretability. Notably, ResNet50 with multi-head attention achieved an accuracy of 97.3%, outperforming those of both the baseline and SE-enhanced models. Moreover, the mean intersection over union (mIoU) for interpretability increased from 75.7% to 82.0%, indicating the model’s improved focus on diagnostically relevant regions.

## 1. Introduction

Cardiovascular diseases (CVDs) are the leading causes of death worldwide according to the World Health Organization (WHO) [1]. The primary method for diagnosing CVDs is the auscultation of heart sounds, using a stethoscope. Heart sounds and murmurs, commonly referred to as phonocardiogram (PCG) signals, provide essential diagnostic information. However, auscultation is a highly complex task that requires extensive expertise and clinical experience. Physicians rely on multiple factors when diagnosing cardiovascular diseases, including the onset of the cardiac cycle, S1 and S2 sounds, and systolic and diastolic intervals. According to these observations, they determine whether the patient has a normal heart sound, systolic murmurs, diastolic murmurs, or both types of murmurs [2].

Phonocardiogram (PCG) recordings can be segmented into individual heartbeat cycles—typically consisting of the S1, systole, S2, and diastole phases—to augment datasets and facilitate more structured analyses [3]. Several automated heart-sound segmentation algorithms have been proposed to identify these components and divide heart-sound signals into cycles, including envelope-based methods [4], hidden Markov models (HMMs) [5], and logistic-regression-HSMM-based algorithms [6,7]. However, in practice, their accuracy can be inconsistent, particularly when applied to noisy or pathological recordings. For instance, envelope-based methods, which rely on amplitude envelopes to infer cardiac boundaries, often miss the true onset or offset of heart sounds because of signal smoothing or distortion. By experimenting with different segmentation techniques, we observed that applying different segmentation techniques yielded varying numbers of segments per sample, reflecting a lack of consistency and robustness across methods. Additionally, the presence of overlapping murmurs or background noise introduces multiple or irregular peaks, which further confound the ability of automated algorithms to distinguish between true cardiac components and artifacts. These limitations reduce the reliability of automatic segmentation. As a result, manually segmented data are essential to ensure the high-quality annotation of heart-sound intervals.

In this study, We used highly accurate manually segmented heart sounds from our dataset HeartWave [8], performed by specialists who carefully listened to each recording and labeled the distinctive features of the heart cycle. The process was meticulous and yielded impressively accurate results. This allowed for the construction of clean and accurate heartbeat cycles, providing a more reliable foundation for training classification models, especially in clinical applications, where interpretability and precision are critical.

Explainable artificial intelligence (XAI) has become increasingly important across several fields of application, particularly in domains where transparency, trust, and accountability are critical—such as transportation, security, medicine, and finance [9]. In the medical imaging field, XAI methods are being actively employed to enhance the interpretability of AI-driven diagnostic systems, thereby improving their trustworthiness and acceptance among healthcare professionals. For instance, XAI has been utilized to clarify the rationale behind decisions made by skin lesion classification systems, offering essential insight into how and why certain predictions are made [10]. These explanations are crucial for justifying the reliability of AI models, especially when their outputs influence high-stakes decisions. More broadly, XAI aims to illuminate the internal mechanisms of complex AI algorithms by generating human-understandable, transparent, and user-friendly outputs [11]. This not only facilitates deeper understanding but also helps in identifying potential biases or artifactual patterns learned during training. As a result, XAI-based approaches are increasingly being integrated into critical domains, such as maritime security, where applications, like ship detection, require both high performance and clear justification for automated decisions [11].

Deep learning has significantly advanced medical diagnostics and patient care [12,13]. However, the “black box” nature of many AI models poses challenges for clinical adoption, as their decision-making processes often lack transparency. Explainable AI (XAI) addresses this issue by providing clear, interpretable insights into model predictions, thereby enhancing trust among healthcare professionals [14]. Techniques like gradient-weighted class activation mapping (Grad-CAM) have been instrumental in visualizing the areas of input data that influence AI decisions [15]. XAI tools, like Grad-CAM, not only enhance interpretability but also provide opportunities to improve model performance by identifying areas where the model’s focus may be misaligned during training.

Because of the scarcity of publicly available heart-sound datasets, particularly those containing a sufficient number of pathological examples, training deep-learning models from scratch becomes highly impractical. This is especially true for rare and difficult-to-diagnose conditions, where data are extremely limited. As a result, the use of pretrained convolutional neural network (CNN) models in transfer learning has become a practical and effective solution for heart-sound and murmur classifications [16].

In recent years, attention mechanisms have been widely integrated into convolutional neural network (CNN) models, contributing to significant performance improvements across various domains [17]. Rather than processing all the input features uniformly, attention enables models to selectively focus on the most informative and relevant regions. This selective weighting helps to replicate a more human-like perception by emphasizing critical cues and suppressing irrelevant information. Attention mechanisms have proven to be particularly effective in tasks such as object detection, object tracking, and re-identification [17].

In our approach, we hypothesize that integrating attention mechanisms into pretrained CNN models can enhance both the classification accuracy and model interpretability. Heart-sound recordings are first manually segmented into complete heartbeat cycles and then transformed into mel-spectrogram images, capturing both time and frequency information. These spectrograms serve as input to the CNN models. Attention mechanisms help the models to focus on the most relevant and informative features, which is especially effective when working with well-defined intervals, such as S1, systole, S2, and diastole. We employ two types of attention separately: squeeze and excitation (SE) attention, which emphasizes important channels in the feature maps, and multihead attention, which captures spatial and contextual dependencies across the spectrogram. By applying these mechanisms to the mel-spectrograms of manually segmented heartbeats, we aim to improve both the diagnostic performance and interpretability critical aspects for clinical trust and adoption. This study makes the following key contributions:

- It leverages manually segmented HeartWave data to develop a classification approach that improves accuracy and interpretability by aligning the model’s decision making with clinically meaningful cardiac phases (S1, systole, S2, and diastole);
- It objectively evaluates the effectiveness of several pretrained CNN models, using manually segmented heartbeat cycles, assessing their performance at clinically meaningful intervals in alignment with established medical practices;
- It integrates two attention mechanisms—squeeze and excitation (SE) and multihead attention—into the pretrained CNN models to enhance the performance and interpretability. These mechanisms guide the models to focus on specifically labeled and diagnostically relevant phases within each heartbeat cycle, rather than treating all the input regions equally, thereby improving both the prediction accuracy and explainability;
- It investigates the impact of dataset balancing on the models’ interpretability by evaluating the mean intersection over union (IoU) of the Grad-CAM visualizations. The results show that the models trained on imbalanced data exhibit reduced interpretability, while balanced datasets produce clearer and more reliable explanations.

The structure of this work is organized as follows: Section 2 presents the existing related studies. Section 3 describes the HeartWave dataset and the manual segmentation process applied. Section 4 introduces explainable AI techniques, with a focus on the Grad-CAM method. The attention mechanisms employed in this study are detailed in Section 5. The pretrained CNN models used are introduced in Section 6.

The proposed methodology, including the experimental setup and evaluation metrics, is outlined in Section 7. The results are presented in Section 8 and discussed in Section 9. Finally, the conclusion is provided in Section 10.

## 2. Related Work

In this section, we explore previous studies relevant to our work. Deep-learning models, particularly convolutional neural networks (CNNs), have shown strong potential in biomedical signal classification [18]. However, these models typically require large-scale datasets for effective training. In the domain of heart-sound and murmur classifications, such datasets are limited, especially for rare and difficult-to-diagnose cardiovascular conditions. To overcome these limitations, researchers have adopted pretrained CNN models using transfer learning, which enables the reuse of features learned from large datasets (e.g., ImageNet) and adapts them to smaller, domain-specific datasets. Automatic segmentation methods that divide heart-sound (PCG) signals into heartbeat cycles have been employed to augment the dataset. Furthermore, attention mechanisms have been integrated into CNN architectures to enhance the model’s performance. By helping the network to focus on the most informative and diagnostically relevant regions of the input (e.g., murmur patterns in spectrograms), attention modules improve both the classification accuracy and model’s efficiency. These methods have been increasingly applied in heart-sound classification, showing promising results in recent studies. Additionally, explainable artificial intelligence (XAI) methods have been increasingly applied to medical imaging to enhance transparency and trustworthiness in AI-driven diagnostic systems. By providing interpretable insights into model predictions, Table 1 summarizes the related studies based on the use of segmentation methods, attention mechanisms, and explainable AI (XAI), along with a comparison to the proposed work.

Wu et al. [22] proposed a deep-learning model, inspired by the squeeze-and-excitation (SE) block attention mechanism, to classify patients’ murmur qualities (harsh vs. blowing) from phonocardiogram (PCG) signals. However, their study does not address model interpretability or explain how the model makes its predictions. In contrast, our work not only applies attention mechanisms (SE and multihead attention) but also integrates Grad-CAM for visual explanation, along with accurate manual segmentation, to enhance both the classification performance and interpretability.

In [23], an attention mechanism was integrated with a CNN model to suppress irrelevant information and focus on crucial, diverse features extracted from heart-sound data. Although this approach is similar to that in our work in leveraging attention to enhance feature representation, their study does not explore explainable AI (XAI) techniques for model interpretation.

Ren et al. [35] proposed a novel attention-based deep-representation-learning method for heart-sound classification. The study integrated an attention mechanism to improve murmur classification and concluded that CNN-based models are among the most effective approaches for this task. However, the work did not explore the model’s interpretability using explainable AI (XAI) techniques.

Yadav et al. [28] proposed a novel attention-based architecture combining a CNN and a BiGRU for abnormal-heartbeat-sound classification. Their findings demonstrated that the proposed model outperformed other deep-learning approaches in terms of the classification accuracy. However, the study did not explore the model’s interpretability or explain how the network arrives at its decisions, an aspect that is central to our work.

In [29], a parallel CNN–Transformer network was proposed to leverage the strengths of both convolutional and attention-based architectures for efficient heart-sound classification. The model achieved strong classification performance, demonstrating the effectiveness of combining local feature extraction with global context awareness (through attention mechanisms). However, the study did not incorporate any interpretability techniques to analyze how the model makes its predictions, an important aspect that is addressed in our work.

Lilhore et al. [19] proposed an attention-based CNN–BiLSTM hybrid model for heart-sound classification. The model employs both spatial attention in a CNN and temporal attention in BiLSTM to focus on the most informative segments in the phonocardiogram (PCG) signals. The architecture effectively captures both local/global features and the temporal context, improving the classification accuracy of complex acoustic signals. The experimental results in PCG and PASCAL heart-sound datasets showed that the proposed model outperformed standard models. However, the study does not incorporate explainable AI (XAI) techniques to interpret the model’s predictions, which distinguishes our approach.

Oliveira [24] explored multimodal learning by developing both an early-fusion model combining 2D CNN backbones for ECG and PCG and a late-fusion model that integrates a 1D CNN for ECG with a 2D CNN for PCG. Their findings demonstrated that multimodal approaches significantly improve the classification performance, with the early-fusion model achieving the best results. Additionally, the study highlighted the potential of incorporating explainable artificial intelligence (XAI) techniques to support clinical decision making by offering transparency about model predictions. Although their focus was on ECG-PCG fusion, our work concentrates solely on PCG signals and places greater emphasis on interpretability through attention mechanisms and Grad-CAM visualization. In [25], a lightweight CNN architecture was proposed for heart-sound classification by integrating multi-feature analysis. The study also employed explainable artificial intelligence (XAI) techniques to interpret the model’s predictions, thereby enhancing the transparency and reliability of the classification process. This aligns with our focus on combining performance with interpretability through attention mechanisms and Grad-CAM visualization.

In [26], heart-sound classification was performed using transfer learning with the OpenL3 network. The system aimed to detect the presence and severity of murmurs without prior segmentation, achieving promising results. The study employed occlusion sensitivity, an explainable artificial intelligence (XAI) technique, to interpret model predictions. The resulting sensitivity maps helped to identify which regions of the input most influenced the model’s decision making, demonstrating the value of XAI in understanding neural network behavior in clinical applications. Although the study successfully demonstrated interpretability, it did not explore the benefits of incorporating multiple attention mechanisms or manual heartbeat segmentation as our work does.

Ren et al. [38] proposed a deep-representation-learning method with an attention mechanism for automatic heart-sound classification. Unlike traditional approaches that rely on handcrafted features, their method learns high-level representations directly from recorded heart-sound data. A key component of the model is the global-attention-pooling layer, which improves performance by estimating the relative contribution of each unit within the feature maps. Although the attention mechanism improved feature representation, the study did not incorporate explainable AI techniques, an area addressed in our work, to ensure model transparency and clinical reliability.

Rajeshwari et al. [39] proposed two clinically explainable frameworks for the automated diagnosis of valvular heart diseases (VHDs), using phonocardiogram (PCG) signals. The first approach combined rule-based models (RBMs) and decision trees (DTs), using clinically relevant features (e.g., duration, intensity, and pitch), although the second used time-frequency representations (TFRs) as input for 2D CNNs and 1D CNNs. A case study on mitral valve prolapse (MVP), with subtypes defined by murmur and systolic click variations, demonstrated the effectiveness of the framework. Notably, PCG segmentation was achieved via a novel TKEO-CWT algorithm, and the use of Grad-CAM for the CNN models provided visual explanations aligning with clinical reasoning. That study highlights the importance of combining high-performance models with clinical interpretability, closely aligned with our approach of integrating attention mechanisms and XAI to enhance model trustworthiness in cardiovascular diagnosis. In [27], an attention-based deep-learning framework was proposed for the detection of heart valve diseases (HVDs), using phonocardiogram (PCG) signals, targeting use in primary healthcare units. Attention mechanisms were integrated at both intra-subband and inter-subband levels to aggregate subband representations effectively, enhancing the diagnostic accuracy. Although the method demonstrated strong generalization and performance, the study did not incorporate explainable AI tools, such as Grad-CAM, which are essential for clinical trust and interpretability, an aspect addressed in our work.

In [34], the authors proposed a simple, yet effective, method for classifying phonocardiogram (PCG) signals, using signal selection techniques combined with transfer learning for cardiovascular disease (CVD) recognition. Among the 17 pretrained CNN architectures evaluated, the VGG19 model achieved the highest performance level. Although the approach effectively streamlines the classification pipeline and improves accuracy, it does not incorporate model interpretability using explainable AI (XAI), an important aspect that is addressed in our work.

Boulares et al. [7] proposed a CVD recognition model that combines supervised and unsupervised machine-learning techniques with convolutional neural networks (CNNs). The method highlights the importance of heart cycle segmentation and segment selection, demonstrating their impacts on performance metrics, such as accuracy, sensitivity, precision, and specificity. Although their study shows strong classification results, it does not address interpretability, an aspect that our research explores through the integration of explainable AI (XAI) techniques.

In [36], the authors proposed a novel deep-learning architecture called DsaNet, which integrates depthwise separable convolution with attention mechanisms for the classification of phonocardiogram (PCG) signals, eliminating the need for complex feature engineering. The study also investigated the effects of different attention modules and data-balancing strategies on the classification performance. DsaNet achieved competitive results compared to those of seven baseline models while maintaining relatively low computational complexity. However, the study did not explore a model that employs explainable AI (XAI) techniques to understand how the model arrives at its predictions, an aspect that is addressed in our work.

Li et al. [20] proposed CAFusionNet, a novel heart-sound classification model that enhances feature representation by fusing features from multiple CNN layers with varying receptive fields. To improve interpretability and highlight relevant diagnostic features, the model integrates channel attention blocks at each layer. Additionally, the study employed homogeneous transfer learning to address the limitation of small datasets. CAFusionNet achieved superior accuracy, outperforming those of traditional deep-learning approaches. Although the study leveraged visualized heatmaps to support feature fusion’s importance, it did not apply dedicated explainable AI (XAI) methods, such as Grad-CAM, as the proposed work does.

Marocchi et al. [31] investigated the fine-tuning of deep convolutional neural networks, including ResNet, VGG, and InceptionV3, on various image representations, such as spectrograms, mel-spectrograms, and scalograms, for heart-sound classification by leveraging both phonocardiogram (PCG) and electrocardiogram (ECG) data. Hidden semi-Markov models (HSMMs) were used as the segmentation method. The study achieved a notable improvement in accuracy. Importantly, the authors also provided interpretability of the model’s learned features, demonstrating clinical relevance. Although the study successfully combines multimodal data and visual representations, it primarily focuses on classification performance, with limited exploration of explainable AI techniques—an aspect our work addresses more comprehensively through Grad-CAM visualizations and attention mechanisms.

In [32], a comprehensive study on time-frequency distributions (TFDs) for heart-sound classification, the authors investigated the effectiveness of various TFDs, such as a continuous wavelet transform (CWT) and a chirplet transform (CT), as inputs to convolutional neural networks (CNNs) for automatic cardiac diagnosis. The findings revealed that transforming heart-sounds to the time-frequency domain significantly outperformed using raw signals. The study also emphasized that increasing the CNN depth and parameters did not necessarily enhance the performance, highlighting the importance of careful architectural design. However, although that work provides valuable insights into CNN optimization and TFD selection, it did not incorporate explainable AI (XAI) techniques to interpret model decisions, an area this current study addresses. Moreover, The segmentation method used divided recordings into fixed five-second segments, which is not always feasible for heart sounds, as PCG signals consist of continuous heartbeat cycles, and such fixed cutting may not accurately align with the actual cardiac events.

Cheng and Sun [33] proposed CTENN, a novel heart-sound classification method that integrates a 1D convolutional module with a transformer encoder. Unlike previous methods, requiring precise segmentation and handcrafted features, CTENN simplifies preprocessing by enabling automatic feature extraction. The model achieved high classification accuracies across three datasets. Although the method demonstrates strong performance in both binary and multiclass tasks, it does not explore model interpretability through explainable AI, which is a key focus of our study. Moreover, the PCG signals were segmented into fixed 2.5 s intervals to capture complete heartbeat cycles. However, this approach may not be medically feasible or accurate, as the segmentation does not necessarily align with the true onset of each heart cycle.

In [30], the authors proposed a novel lightweight architecture for congenital heart disease (CHD) detection using a hybrid model that combines 2D convolutional neural networks (CNNs), bidirectional long short-term memory (Bi-LSTM), and an attention mechanism. The model was designed to classify pediatric phonocardiogram (PCG) recordings into five categories, including normal, functional, pathological, ventricular septal defect (VSD), and atrial septal defect (ASD), achieving a high accuracy rate. The study represents the first deep-learning-based approach for classifying pediatric heart sounds into more than four diagnostic categories. Despite its promising results and low computational complexity, the study does not incorporate explainability techniques, such as Grad-CAM, to interpret model decisions, an aspect that is a central focus of our work.

In [37], a deep-learning framework was proposed to diagnose exercise-induced cardiac fatigue (EICF), a condition that can lead to myocardial damage or sudden cardiac events because of high-intensity physical activity. The authors introduced a novel approach using heart-sound signals to reflect cardiac inotropy, addressing the limitations of traditional, expensive diagnostic tools. A heart-sound dataset was collected from 20 subjects and segmented using a discrete wavelet transform and a hidden semi-Markov model. The study then employed a custom network that integrates residual mapping with attention mechanisms to enhance the recognition accuracy. The proposed model achieved outstanding results, demonstrating the potential of heart-sounds as a reliable and non-invasive diagnostic alternative for EICF. Although the model incorporated attention mechanisms to improve the classification performance, it did not employ explainable AI (XAI) techniques to interpret how decisions were made, an aspect addressed in our work through Grad-CAM-based visualizations.

In [21], the authors proposed ENACT–Heart, an ensemble framework combining vision transformers (ViTs) and convolutional neural networks (CNNs) within a mixture of experts (MoEs) architecture for heart-sound classification. The ensemble approach achieved a high classification accuracy rate, outperforming those of individual ViT and CNN models. This highlights the effectiveness of combining transformer-based and convolutional features for cardiovascular-sound analysis. However, the study did not incorporate explainable AI (XAI) methods to interpret the model’s decision-making process, an aspect addressed in our work. In addition to this, as demonstrated in [33], the approach of segmenting recordings into fixed five-second intervals may not be suitable for heart-sound analysis, because PCG signals represent continuous heartbeat cycles, and such arbitrary segmentation may fail to accurately correspond with the true cardiac events.

In summary, although previous studies have examined the CNN-based classification of heart sounds, most rely on automated segmentation methods or simple fixed-length audio splitting, often lacking interpretability of the model’s predictions. To bridge this gap, our study explores the use of pretrained CNN architectures applied to manually segmented heart-sound data, enriched with squeeze-and-excitation (SE) and multihead attention mechanisms. Additionally, we employ Grad-CAM to visualize and interpret the model’s decisions. By assessing both performance metrics and interpretability, this work offers valuable insights into how attention mechanisms and segmentation quality jointly impact classification accuracy and the clinical trustworthiness of predictions.

## 3. Annotation and Manual Segmentation of the HeartWave Dataset

The HeartWave dataset [8], developed through a collaboration between King Abdulaziz University and three hospitals, stands as one of the most extensive and comprehensive collections of heart-sound recordings. It comprises 1353 PCG recordings, each labeled at the recording level, providing a valuable diagnostic context. One of the most notable features of the dataset is the inclusion of murmur grades ranging from 1 to 6, determined using echocardiography, which reflect the severity and acoustic characteristics of heart murmurs in real-world clinical scenarios. All the recordings are stored in .wav format, with an average duration of 21.57 s per recording. The dataset covers nine classes, including normal heart sounds and eight types of heart-murmur-related cardiovascular diseases. These include both common conditions and rare or diagnostically complex ones, such as pulmonic stenosis, tricuspid stenosis, and tricuspid regurgitation, which are often challenging for physicians to diagnose through auscultation alone. The HeartWave dataset was carefully labeled and annotated by an expert team of cardiologists to ensure high-quality and clinically accurate annotations. Table 2 summarizes the number of recordings and their average durations for each class in the dataset.

### 3.1. Clinical Workflow of Auscultation-Based Diagnosis

In routine clinical practice, the diagnosis of cardiovascular abnormalities through auscultation follows a well-established clinical workflow [40]. Physicians begin by reviewing the patient’s history and reported symptoms to guide their initial diagnostic hypotheses. This is followed by a physical examination, during which the patient is positioned appropriately—sitting, supine, or in the left lateral decubitus position—to optimize the audibility of specific heart sounds. Using a stethoscope, clinicians systematically auscultate the four primary cardiac landmarks: the aortic, pulmonic, tricuspid, and mitral areas. At each site, they carefully interpret the sequence of heart sounds across a complete cardiac cycle, identifying key components, such as the first heart sound (S1), systole, second heart sound (S2), and diastole. The S1 sound is particularly important, as it marks the beginning of ventricular systole and serves as a timing anchor for identifying and classifying murmurs. It provides valuable diagnostic insight into the function of the mitral and tricuspid valves, and changes in its intensity or timing can indicate conduction abnormalities or structural heart disease. If murmurs are present, they are further assessed based on timing (systolic, diastolic, or continuous), pitch, quality, intensity (graded on a scale from 1 to 6), anatomical location, radiation, and their response to physiological maneuvers. Finally, auscultatory findings are documented and integrated with other diagnostic data to arrive at a comprehensive and clinically informed diagnosis.

### 3.2. Annotation of the HeartWave Dataset

The HeartWave dataset was created through a collaborative effort between King Abdulaziz University and three medical institutions: the National Heart Institute in Cairo, Egypt; King Abdulaziz Specialist Hospital in Taif, KSA; and King Faisal Medical Complex in Taif, KSA [8]. To ensure clinically accurate and high-quality labels for effective cardiovascular disease classification, a rigorous annotation protocol based on patients’ echocardiogram reports was employed.

Each echocardiogram report provided comprehensive details regarding the patient’s cardiac structure, function, and any observed abnormalities. A team of experienced cardiologists carefully examined these reports and supervised the entire annotation process. They reviewed the clinical descriptions of pathological findings and cross-referenced them with the corresponding heart-sound recordings. According to their expert interpretation, echocardiographic findings, and clinical judgment, they assigned precise labels to the heart sounds, indicating the presence or absence of specific cardiac diseases or conditions.

This meticulous process ensured that all the dataset labels were accurate, consistent, and validated in accordance with echocardiographic diagnoses.

### 3.3. Expert-Guided Manual Segmentation of the HeartWave Dataset

The HeartWave dataset was manually segmented, with each recording carefully reviewed by a team of medical experts. This manual segmentation was conducted in collaboration with Sparrow BioAcoustics [41], a company specializing in clinical-grade cardiac signal technologies. The segmentation process was thoroughly assessed by Sparrow BioAcoustics’ expert medical team. Each recording was independently evaluated by at least three medical professionals and finalized by a senior clinical manager to ensure the highest quality of annotation and segmentation. Experts precisely identified the intervals corresponding to key components of the heartbeat cycle, including S1, S2, systole, and diastole. In parallel, each recording was also examined to determine the presence of systolic murmurs, diastolic murmurs, combined murmurs (systolic and diastolic), or segments that were unsegmentable because of excessive noise or signal ambiguity. This rigorous process ensured that the dataset reflects clinically meaningful events with high annotation quality.

**Figure 1:**
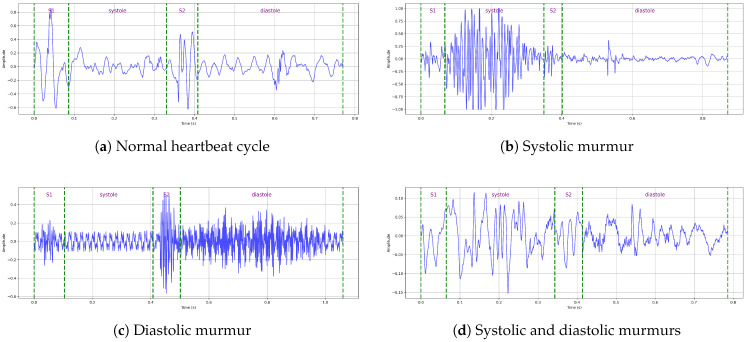
Examples of manually annotated heartbeat cycles from the HeartWave dataset. Each waveform is annotated, with vertical dashed lines indicating the start and end times of the four cardiac phases: S1, systole, S2, and diastole, along with whether the heartbeat cycle is normal or contains murmurs: (**a**) A normal heartbeat cycle, (**b**) a heartbeat cycle with a systolic murmur, (**c**) a heartbeat cycle with a diastolic murmur, and (**d**) a heartbeat cycle with both systolic and diastolic murmurs.

This manual segmentation process significantly enhances the dataset’s quality by filtering out unclear intervals and providing clean, clinically validated annotations. As a result, the refined HeartWave dataset contains a total of 30,562 heartbeat cycles, distributed as follows: normal, 13,905 cycles; systolic murmurs, 10,849 cycles; diastolic murmurs, 1625 cycles; and systolic and diastolic murmurs, 4183 cycles, as shown in Table 3. This expert-guided segmentation provides a reliable foundation for both training and evaluating machine-learning models, particularly in clinical applications, where accurate interpretation is critical. Figure 1 presents examples of manually annotated heartbeat cycles from the HeartWave dataset. Each waveform is annotated with vertical dashed lines marking the start and end of the four cardiac phases: S1, systole, S2, and diastole. The annotations also indicate whether the heartbeat cycle is normal or contains murmurs, specifically, (a) a normal heartbeat cycle with no murmurs, (b) a cycle with a systolic murmur, (c) a cycle with a diastolic murmur, or (d) a cycle exhibiting both systolic and diastolic murmurs. These examples demonstrate the clinical richness and clarity of the manual segmentation process applied in this study.

Because the manual segmentation process of the HeartWave dataset was still ongoing during the initial stages of our study, we conducted our experiments using a subset of the dataset. For the balanced dataset experiments, we selected 237 samples per class, ensuring equal representation across the four categories: normal, systolic murmurs, diastolic murmurs, and systolic–diastolic murmurs (referred to as abnormal). For the imbalanced dataset experiment, we used a distribution that reflects the natural class imbalance present in clinical scenarios: 2000 samples of normal, 1500 of systolic murmurs, 237 of diastolic murmurs, and 1000 of systolic–diastolic murmurs. The balanced dataset was used to evaluate the performances of pretrained CNN models in three configurations: baseline, with squeeze-and-excitation (SE) attention, and with multihead attention. The imbalanced dataset, on the other hand, was specifically utilized to investigate the impact of data balancing on the model’s interpretability. This setup allowed us to explore and compare the model’s performances under both balanced and imbalanced conditions, as shown in Table 4.

## 4. Explainable Artificial Intelligence (XAI)

Artificial intelligence (AI) has become increasingly prevalent in both medical research and clinical applications, particularly within the domain of medical imaging [42]. Deep learning, a subset of AI, has shown remarkable success in tasks such as image classification, segmentation, and object detection. These models autonomously learn feature hierarchies from data, starting from basic elements, like edges, and evolving toward more abstract and complex features, for instance, the irregular contours of tumors, which enable them to make high-level diagnostic decisions [43]. Despite their success, the internal workings of deep-learning models are often difficult because of their intricate architectures, composed of multiple nonlinear layers. This complexity makes it challenging to establish how specific predictions are formed, earning them the reputation of being “black-box” systems [44]. This lack of transparency poses significant risks, especially in healthcare settings, where unexplained or biased outcomes can directly impact patient safety and treatment decisions. Although significant efforts have been made to maximize models’ accuracy and robustness, these gains frequently come at the cost of interpretability. As models become deeper and more parameter rich, understanding their logic becomes increasingly elusive. This presents a major barrier in clinical adoption, where interpretability is essential to earn the trust of physicians, patients, and regulatory authorities. In response, explainable artificial intelligence (XAI) has emerged as a critical field focused on explaining the decision-making process of AI models [42]. XAI methods aim to make model predictions more transparent and understandable, offering insights that help users to grasp why a particular decision was made. These techniques generally fall into two categories: model-agnostic approaches, which are applicable to a variety of algorithms, and model-specific techniques designed for particular neural architectures. By making AI outputs more interpretable, XAI facilitates clinical validation and fosters confidence in automated diagnostics [42].

### Gradient-Weighted Class Activation Mapping (Grad-CAM)

Gradient-weighted class activation mapping (Grad-CAM) is a powerful interpretability technique designed to provide visual insights into the inner workings of convolutional neural networks (CNNs). It does so by generating a class-specific heatmap that highlights the regions of an input image or signal that most strongly influences the model’s prediction. This visualization is especially valuable in tasks such as classification, where understanding the model’s focus is crucial. Grad-CAM enhances the transparency of deep-learning systems by revealing which parts of the input are the most relevant to a particular decision. This is achieved by leveraging the gradients of the target class flowing into the last convolutional layer and weighting the corresponding feature maps to identify and visualize areas of high importance [45].

The process begins when a convolutional neural network (CNN) receives an input image, which is propagated through multiple convolutional and pooling layers. These layers extract hierarchical features at increasing levels of abstraction, ultimately producing high-level feature maps in the final convolutional layer.

Let $A^{k}$ represent the *k*th feature map generated in this final convolutional layer. The class score ($y^{c}$), which indicates the model’s confidence in predicting class *c*, is calculated based on these feature maps, typically through fully connected layers that follow. To assess the influence of each feature map ($A^{k}$) on the score ($y^{c}$), Grad-CAM computes the gradient of $y^{c}$ with respect to $A^{k}$, using backpropagation, as follows:

(1) $$\frac{\partial y^{c}}{\partial A^{k}}$$

This gradient captures how sensitively the output score ($y^{c}$) changes with respect to changes in the activation map ($A^{k}$), identifying which regions of the input significantly impact the model’s decision.

To quantify the relative importance of each feature map ($A^{k}$) for predicting class *c*, Grad-CAM computes neuron importance weights ($\alpha_{k}^{c}$). These weights are obtained via global average pooling over the gradients as follows:

(2) $$\alpha_{k}^{c}=\frac{1}{Z}\sum_{i}\sum_{j}\frac{\partial y^{c}}{\partial A_{ij}^{k}}$$

were $A_{ij}^{k}$ denotes the activation at spatial position (i,j) in the *k*th feature map, and *Z* is the total number of spatial locations (i.e., pixels) in $A^{k}$. The scalar ($\alpha_{k}^{c}$) thus reflects the overall impact of the feature map ($A^{k}$) on the prediction for class *c*.

Next, these weights ($\alpha_{k}^{c}$) are used to compute a class-specific localization map ($L_{\mathrm{Grad}-\mathrm{CAM}}^{c}$) by performing a weighted sum over the feature maps as follows:

(3) $$L_{\mathrm{Grad}-\mathrm{CAM}}^{c}=\mathrm{ReLU}\left(\sum_{k}\alpha_{k}^{c}A^{k}\right)$$

ReLU activation ensures that only those features contributing positively to the class prediction are preserved, by zeroing out negative influences. The resulting localization map ($L_{\mathrm{Grad}-\mathrm{CAM}}^{c}$) is then upsampled to match the spatial resolution of the original input image. This produces a heatmap that visually highlights the most relevant regions in the input that influenced the prediction for class *c* [45].

In this study, we employed gradient-weighted class activation mapping (Grad-CAM) to improve the interpretability of our pretrained CNN models trained on mel-spectrogram images. Grad-CAM offers significant advantages, particularly in medical image analysis, where transparent and human-interpretable explanations are critical. It generates intuitive visualizations by highlighting the regions of the input that contribute the most to the model’s predictions, thereby enhancing both trust and understanding. A key benefit of Grad-CAM is its model-agnostic nature, enabling it to be applied to any CNN-based architecture without requiring structural changes [15]. By localizing class-discriminative regions, Grad-CAM not only supports interpretability but also serves as a powerful diagnostic tool. It enables researchers to observe and refine the model’s behavior during training, helping to optimize its performance and reduce misclassifications.

## 5. Attention Mechanism

The attention mechanism was originally introduced within sequence-to-sequence models for machine translation using an encoder–decoder framework [46]. It was designed to dynamically capture and summarize context-relevant information across varying input sequence lengths. The development of self-attention further refined this concept by allowing models to focus on internal dependencies within a single input, making it particularly effective for capturing long-range relationships. In the domain of computer vision, attention mechanisms are frequently employed to enhance convolutional neural networks (CNNs) by enabling the model to concentrate on the most informative regions of the input. This selective focus improves the classification performance by guiding the network toward features that are the most relevant for a given task. Specifically, attention mechanisms evaluate spatial correlations across distant pixels by assigning importance weights, effectively highlighting discriminative regions within an image. In the contexts of heart-sound classification and murmur detection, interpretability is critical. It is essential to identify which segments of the visualized input (e.g., a mel-spectrogram) are the most relevant for diagnoses. Attention mechanisms fulfill this need by automatically learning to highlight and focus on diagnostically meaningful regions. In this study, we incorporate two types of attention modules: multihead attention and squeeze-and-excitation (SE) attention, both of which are integrated into pretrained CNN-based models to enhance their performance and interpretability.

### 5.1. Multihead Attention

**Figure 2:**
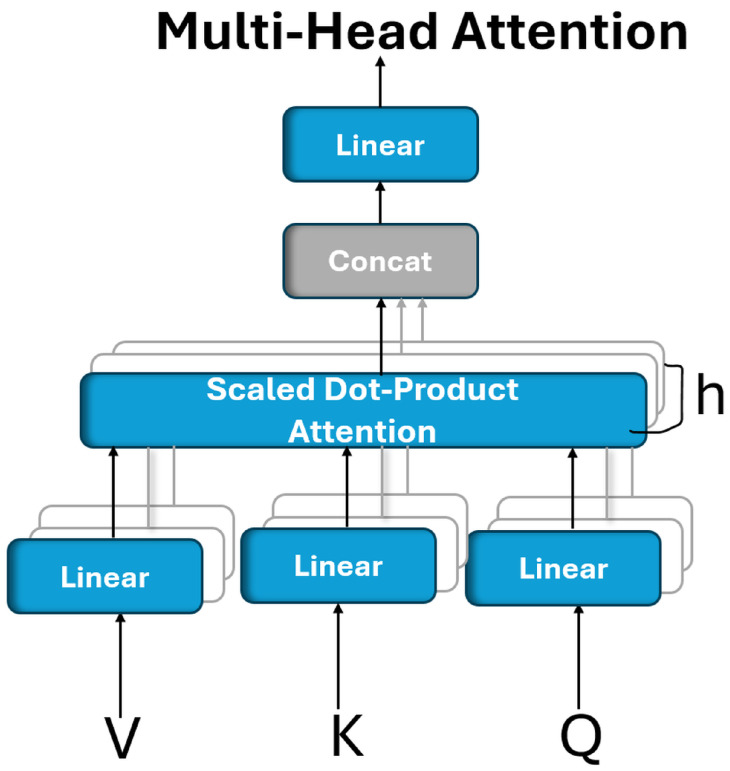
Multihead attention is composed of multiple attention layers operating simultaneously in parallel.

An attention mechanism operates by associating a given query with a set of key–value pairs to produce an output, where all the components (query, keys, values, and output) are vector representations. The output is calculated as the weighted sum of the values, where the weights reflect the relevance (or compatibility) between the query and each key determined through a similarity or scoring function [46]. To enhance the learning capacity and enable the model to focus on different aspects of the input, the multihead attention mechanism was introduced. Rather than using a single attention computation over full-dimensional vectors, this approach splits the attention process into multiple independent “heads”. Each head linearly projects the queries, keys, and values into lower-dimensional subspaces, using learned transformation matrices. The attention is then computed in parallel across these subspaces, allowing the model to capture diverse relationships and dependencies. The outputs of all the heads are then concatenated and passed through another linear transformation to produce the final attention output, as shown in Figure 2 [46].

Multihead attention enables a model to simultaneously focus on different types of information from various representation subspaces. Unlike single-head attention, which computes a single context vector, multihead attention performs multiple attention operations in parallel, allowing the model to capture more nuanced dependencies from different positions in the input. Formally, multihead attention is defined as follows:

(4) $${\mathrm{head}}_{i}=\mathrm{Attention}\left(QW_{i}^{Q},KW_{i}^{K},VW_{i}^{V}\right)$$

where each attention head is computed as follows:

(5) $${\mathrm{head}}_{i}=\mathrm{Attention}\left(QW_{i}^{Q},KW_{i}^{K},VW_{i}^{V}\right)$$

where the projection matrices are defined as follows:

$W_{i}^{Q}\in R^{d_{\mathrm{model}}\times d_{k}}$, $W_{i}^{K}\in R^{d_{\mathrm{model}}\times d_{k}}$, $W_{i}^{V}\in R^{d_{\mathrm{model}}\times d_{v}}$, and the output projection matrix ($W^{O}\in R^{hd_{v}\times d_{\mathrm{model}}}$). These matrices are learned during training [46].

In this study, multihead attention (MHA) was employed because of its ability to simultaneously capture diverse types of feature relationships and spatial dependencies within the input representation. It provides multiple parallel attention heads, each capable of learning distinct patterns or attending to different regions of interest.

### 5.2. Squeeze-and-Excitation (SE) Attention Mechanism

The squeeze-and-excitation network (SENet), introduced in 2017, aims to enhance the representational capacity of convolutional neural networks by explicitly modeling the interdependencies between feature channels [47]. SENet is known for its simplicity and modularity, making it highly adaptable for integration into existing architectures. Its main objective is to allow the network to focus on the most informative feature channels by capturing global contextual information.

**Figure 3:**
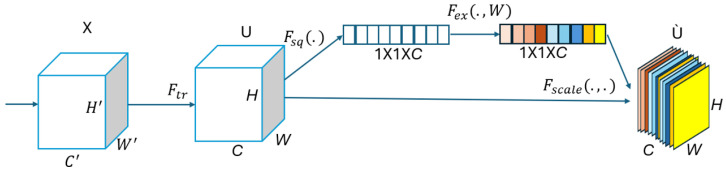
A squeeze-and-excitation block.

The SENet module operates through three sequential steps: **squeezing**, **exciting**, and **reweighting**, which are applied after the convolutional operation ($F_{\mathrm{tr}}$), as illustrated in Figure 3.

In the Squeezing step, a global average pooling operation is applied to each individual feature map (*U*), reducing it to a 1×1×C-dimensional channel descriptor via the squeeze function ($F_{\mathrm{sq}}\left(\cdot \right)$) as follows:

(6) $$z_{c}=F_{\mathrm{sq}}\left(U_{c}\right)=\frac{1}{H\times W}\sum_{i=1}^{H}\sum_{j=1}^{W}U_{c}\left(i,j\right)$$

where $z_{c}$ denotes the squeezed scalar descriptor for the *c*th channel, as obtained via global average pooling. $F_{\mathrm{sq}}$ is the squeeze function, and $U_{c}\left(i,j\right)$ represents the value at spatial position (i,j) in the *c*-th feature map. *H* and *W* are the height and width of the feature map. This operation compresses spatial information to a single representative value per channel, capturing its global contextual information.

In the Excitation step, a self-gating mechanism, composed of two fully connected layers and a sigmoid activation function, is used to learn nonlinear channel-wise dependencies. This produces a set of weights for each channel, using the excitation function ($F_{\mathrm{ex}}\left(\cdot ,w\right)$) as follows:

(7) $$s=F_{\mathrm{ex}}\left(z,W\right)=\sigma \left(W_{2}\;\delta \left(W_{1}z\right)\right)$$

In Equation (7), *s* represents the learned channel-wise excitation weights, obtained by passing the squeezed descriptor ($z\in R^{C}$) through two fully connected (FC) layers. $W_{1}\in R^{\frac{C}{r}\times C}$ and $W_{2}\in R^{C\times \frac{C}{r}}$ are the weight matrices of the two FC layers, where *r* is the reduction ratio controlling the bottleneck’s dimensionality. The function δ(·) denotes the ReLU activation, and σ(·) is the sigmoid function, which constrains the output to the range [0,1]. This operation enables the model to capture nonlinear inter-channel dependencies and assign importance weights to each feature map channel. Also, δ denotes the ReLU activation, and σ is the sigmoid function.

The final step reweights each channel of the input feature map, using the learned excitation vector:

(8) $${\tilde{U}}_{c}=F_{\mathrm{scale}}\left(U_{c},s_{c}\right)=s_{c}\cdot U_{c}$$

where ${\tilde{U}}_{c}$ denotes the recalibrated output of the *c*th feature map after being scaled by its corresponding excitation weight ($s_{c}$). The function $F_{\mathrm{scale}}$ defines the element-wise multiplication between the input feature map and the learned importance weights. This mechanism enables the network to emphasize the most critical channels while suppressing those that are less informative, thereby improving both the feature learning and overall model performance [17].

In this study, we employed the squeeze-and-excitation (SE) block because of its simplicity, negligible impact on computational complexity, and its ease of integration into any pretrained CNN architecture. Its incorporation enhances the model’s ability to focus on informative features, leading to improved classification performance.

## 6. Pretrained CNN Models

Pretrained CNN models are deep convolutional neural network architectures that have been trained in large-scale datasets, such as ImageNet. These models learn robust and generalizable feature representations from millions of labeled images, capturing essential visual patterns that can be applied across a wide range of tasks [48]. Transfer learning adapts these pretrained models to a new, often related, task by transferring their parameters to a target dataset, thereby addressing the challenges associated with limited training data [48]. In our study, we leverage six pretrained CNN models: ResNet50, ResNet152, MobileNet, MobileNetV2, VGG19, and EfficientNetV2B0 and fine-tune them for a heart-sound classification task. We will explore the architectures of each model in detail.

### 6.1. ResNet50

**Figure 4:**
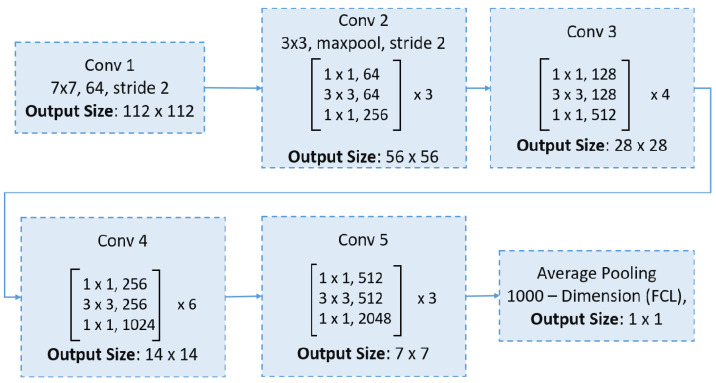
An overview of the ResNet50 architecture.

ResNet introduced the concept of residual blocks with skip connections, which facilitate more efficient gradient propagation during training by bypassing one or more layers [49]. These shortcut connections provide a direct path for gradients, mitigating the vanishing gradient problem. Moreover, some variants of ResNet employ a preactivation design, where batch normalization and ReLU activation are applied before the convolutional layers, further stabilizing the training process. This innovative architecture has revolutionized deep learning by enabling the training of very deep networks with hundreds of layers. ResNet50, a variant featuring 50 weight layers, leverages these residual blocks and skip connections, striking a favorable balance between network depth and accuracy, which has contributed to its widespread use in image classification tasks. The ResNet50 network begins with a 7 × 7 convolution and max pooling, followed by four stages of residual bottleneck blocks (with 3, 4, 6, and 3 blocks, respectively), and concludes with global average pooling and a fully connected layer for classification, as shown in Figure 4.

### 6.2. ResNet152

**Figure 5:**
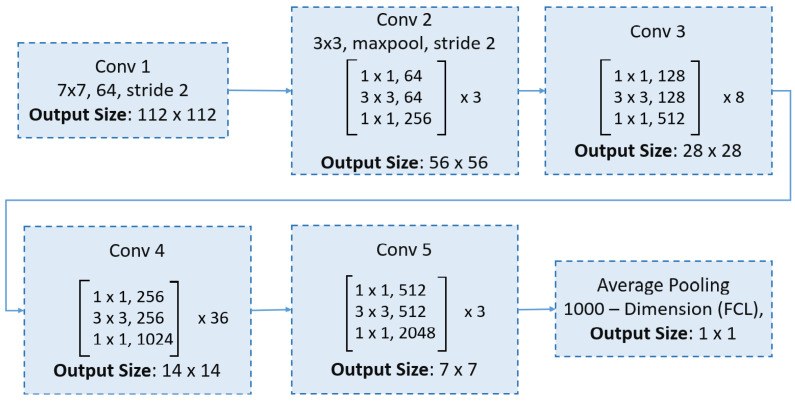
An overview of the ResNet152 architecture.

ResNet152 is a deeper variant of the ResNet architecture, comprising 152 layers [49]. Its additional depth enables the network to capture more complex and nuanced patterns in the data, making it particularly suitable for applications where top-tier accuracy is essential. The ResNet152 architecture begins with an initial convolutional layer, followed by five stages of residual blocks that incorporate bottleneck layers, as demonstrated in Figure 5.

### 6.3. MobileNet

**Figure 6:**
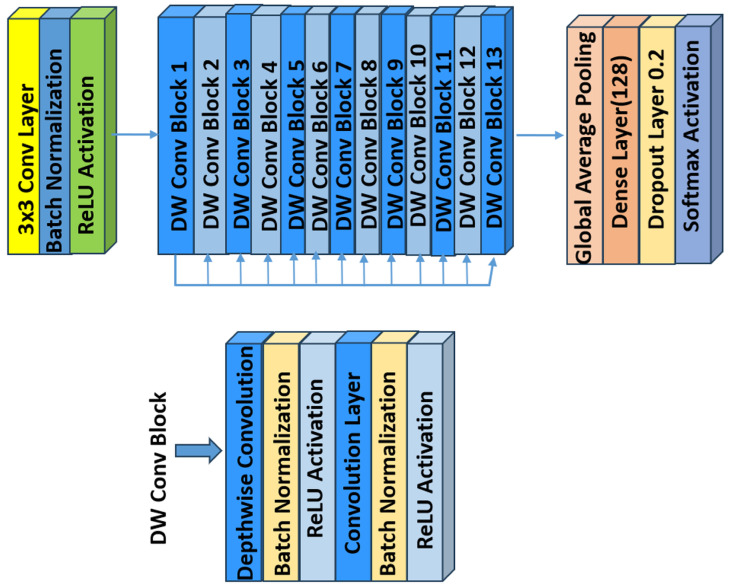
An overview of the MobileNet architecture.

MobileNet models are built on a streamlined architecture that employs depthwise separable convolutions. These convolutions break down a standard convolution into two separate operations: a depthwise convolution that applies one filter per input channel, and a pointwise convolution (1 × 1 convolution) that combines these outputs. This separation significantly reduces computational costs and the number of parameters, enabling the construction of lightweight, yet effective, deep neural networks [50]. Figure 6 illustrates the architecture of MobileNet.

### 6.4. MobileNetV2

**Figure 7:**
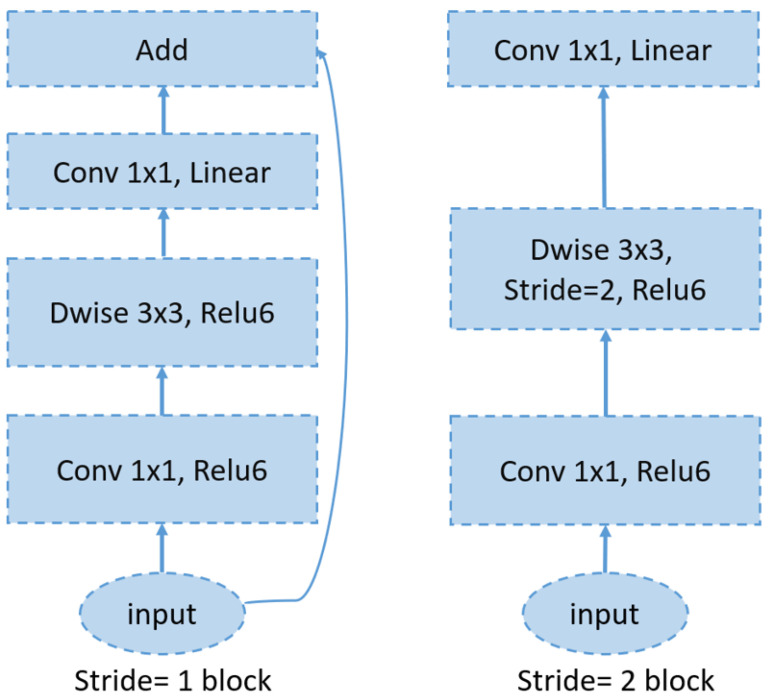
An overview of the MobileNetV2 architecture.

MobileNetV2 is a convolutional neural network architecture designed for efficient performance on mobile devices. It is built around an inverted residual framework, where skip connections are established between bottleneck layers to facilitate effective feature reuse (see Figure 7). Within each bottleneck, an expansion layer employs lightweight depthwise convolutions to introduce nonlinearity and refine the feature extraction. Overall, MobileNetV2 starts with an initial fully convolutional layer comprising 32 filters, followed by 19 residual bottleneck layers. It employs ReLU6 as the activation function, uses a 3 × 3 kernel size, and incorporates both dropout and batch normalization techniques to enhance regularization and training stability [51].

### 6.5. VGG19

**Figure 8:**
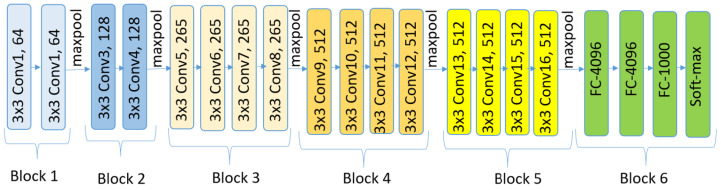
An overview of the VGG19 architecture.

VGG [52], which stands for visual geometry group, represents a family of convolutional neural network (CNN) architectures designed for image classification. Two key characteristics of VGG networks are their simplicity and uniformity, achieved using small (3×3) convolutional filters consistently throughout the architecture. VGG19, in particular, is an extended variant of the VGG architecture, comprising 19 weight layers compared to the 16 layers in VGG16. Like VGG16’s design, its design is straightforward, involving the stacking of multiple 3×3 convolutional layers interleaved with max-pooling layers to progressively reduce the spatial dimensions. However, VGG19 incorporates three additional convolutional layers, resulting in increased depth that enables the capture of more complex features from the input data. This enhanced depth can be advantageous for certain tasks. Figure 8 shows the VGG19 architecture.

### 6.6. EfficientNetV2BO

**Figure 9:**
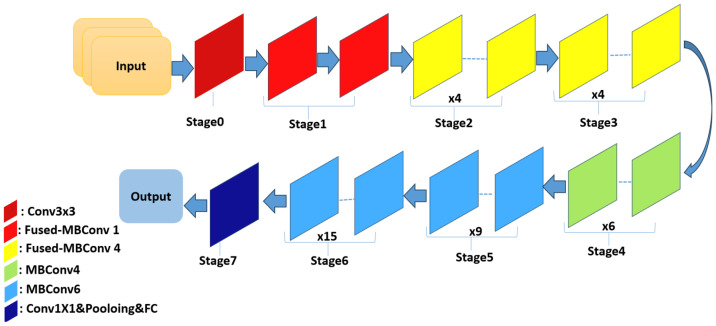
The EfficientNetV2-S architecture.

EfficientNetV2 is a novel family of convolutional networks designed to achieve faster training and superior parameter efficiency compared to those of its predecessors. It represents an improved iteration of EfficientNet, with the primary goal of optimizing both the training velocity and parameter utilization. In previous network layers, the standard depthwise convolutional operations (MBConvs) were found to be slow on modern hardware, despite their lower parameter count relative to those of traditional convolution models. To overcome this limitation, EfficientNetV2 integrates a combination of MBConv and Fused-MBConv layers, which accelerates the training process without increasing the overall parameter count. EfficientNetV2B0 is one of the variants introduced by Tan and Le [53]. Figure 9 shows the EfficientNetV2-S architecture.

In this study, these pretrained CNN models were adopted because of their demonstrated effectiveness and extensive use in medical imaging tasks [54]. Table 5 summarizes the selected pretrained architectures, including their depth, model size, and total number of parameters.

## 7. Proposed Methodology

**Figure 10:**
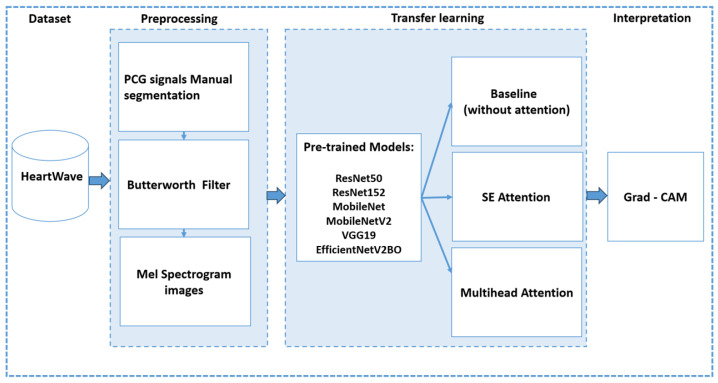
An overview of proposed methodology.

This section details our proposed approach, which consists of three main stages: (1) preprocessing, (2) transfer learning using pretrained CNN models both with and without integrated attention mechanisms, and (3) interpretation of model predictions using Grad-CAM (see Figure 10).

### 7.1. Preprocessing

**Figure 11:**
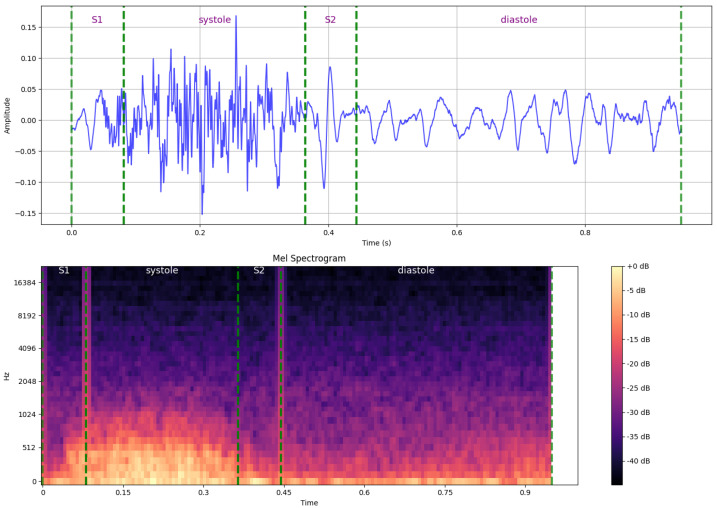
An example of converting a single heartbeat cycle, which demonstrates a systolic murmur, from its original time-domain waveform to a mel-spectrogram (time-frequency domain), with annotations indicating key cardiac phase boundaries.

In this stage, the Heartwave dataset is manually annotated and segmented by experts, as discussed in Section 3. The PCG signals are then denoised using a fourth-order low-pass Butterworth IIR filter, with a cutoff frequency set at 600 Hz. This filtering step isolates crucial frequency components for each heartbeat cycle. Next, the filtered signals are transformed to the time-frequency domain through spectrogram generation, and the resulting images are used for classification and interpretation. Specifically, the short-time Fourier transform (STFT) is employed to convert the one-dimensional PCG signals to 224 × 224 pixel mel-spectrograms. For all the heart-sound samples in this study, a Hann window with a length of 1024 and a hop length of 256 was used, producing mel-spectrograms with 40 mel bands, which effectively capture both the high-frequency and low-frequency components of the signal. Figure 11 provides an example of converting a single heartbeat cycle, which contains a systolic murmur, from its original time-domain waveform to a mel-spectrogram. The top plot illustrates the PCG waveform, with vertical dashed lines indicating the boundaries of S1, systole, S2, and diastole, while the bottom plot shows the corresponding mel-spectrogram.

### 7.2. Transfer-Learning-Pretrained CNN Models

**Figure 12:**
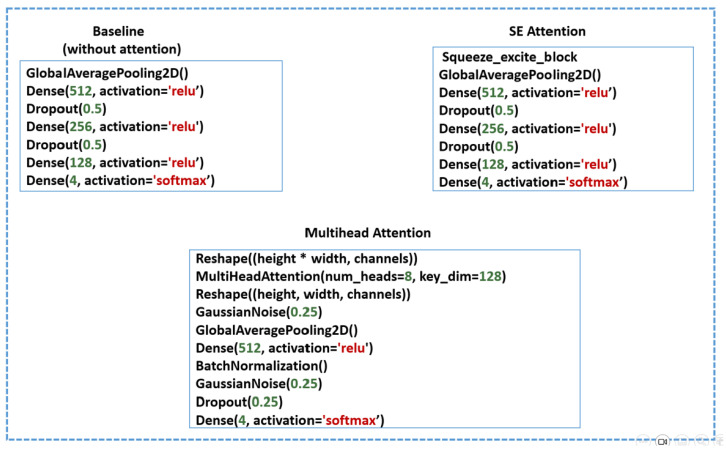
Architectural configurations of the three evaluated methods: Baseline, Squeeze-and-excitation (SE) attention, and Multihead attention.

In this stage, we fine-tune the pretrained CNN models (ResNet50, ResNet152, MobileNet, MobileNetV2, VGG19, and EfficientNetV2B0) to adapt them for heart-sound and murmur classifications. We conducted four experiments using three methods to evaluate the impacts of the attention mechanisms on the performance and interpretability. For all the methods, the pretrained model weights are frozen. The architectural configurations of each method are illustrated in Figure 12, highlighting the specific components used in each approach.

In the baseline (no attention) approach, four layers are appended: a global-average-pooling (GAP) layer to reduce overfitting by minimizing the number of trainable parameters while preserving essential spatial features, followed by three dense layers with ReLU activation to capture high-level, nonlinear representations necessary for accurate classification. Dropout layers are incorporated between the dense layers to serve as a regularization technique. Finally, a softmax layer is used for multiclass classification.

In the SE attention method, a squeeze-and-excitation (SE) block is applied to recalibrate and emphasize the most informative feature channels. This is followed by the same appended layers used in the baseline approach.

In the multihead attention method, the feature maps are first reshaped and processed using an eight-head multihead attention mechanism with a key dimension of 128, allowing the model to attend to different spatial regions and capture diverse contextual information. The attention output is then reshaped back to its original dimensions, followed by the addition of a Gaussian noise layer for regularization, a GAP layer, dense layers, and batch normalization to accelerate convergence and stabilize the training process. The final multiclass classification is performed using a softmax layer.

In the fourth experiment, to investigate the impact of data balancing on the model’s interpretability, we apply the baseline approach using MobileNetV2, as it is the most representative model in terms of interpretability in the imbalanced dataset.

The pretrained CNN models were fine-tuned using a consistent set of hyperparameters (See Table 6). The input image size is (224 × 224) pixels.The dataset was split into 70% for training, 15% for validation, and 15% for testing. A batch size of five was used during the training, with a learning rate of 0.0001 and the Adam optimizer. Early stopping was implemented based on the validation categorical accuracy, with a patience of 10 epochs, and the best model weights were restored upon termination.

### 7.3. Interpreting

In the interpretation stage, we aimed to assess how well the models focused on clinically relevant regions by applying Grad-CAM to true positive predictions. For each class in the test dataset, ten true positive samples were selected. Each trained model was then reloaded, and Grad-CAM was applied to generate class-specific activation heatmaps.

To evaluate the accuracy of these visual explanations, we utilized annotated cardiac-phase segmentation—created using the LabelMe tool—to draw the ground truth’s bounding boxes for each sample. Predicted activation regions from Grad-CAM were also extracted and overlaid as predicted bounding boxes. We then calculated the intersection over union (IoU) for each example by comparing the predicted and ground truth’s bounding boxes. The average mean IoU (mIoU) per class was subsequently computed. For the normal and abnormal (systolic–diastolic) classes, where two distinct cardiac phases (S1 and S2 in normal and systolic and diastolic in abnormal) are annotated, we computed the mean IoU as the average across both regions. At the end of the evaluation, the mean IoU (mIoU) was averaged across all the classes to obtain an overall interpretability score for each model.

The interpretation procedure followed these steps:

1. Select 10 true positive examples from each class;
2. For each sample, plot the annotated cardiac phases (S1, S2, systolic, and diastolic);
3. Load each of the six trained models and apply Grad-CAM to the last convolutional layer;
4. Draw the ground truth’s bounding boxes based on the manually annotated segments using the LabelMe tool;
5. Overlay the predicted activation bounding boxes generated from Grad-CAM on the same mel-spectrogram image;
6. Calculate the intersection over union (IoU) for each example, using Equation (11);
7. Compute the average IoU per class For the classes with two cardiac phases (e.g., normal and abnormal), calculate the mean of the two IoUs.

**Figure 13:**
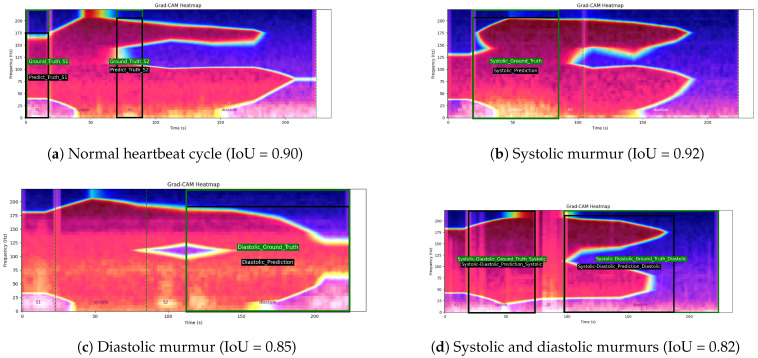
Examples of Grad-CAM heatmaps generated using the fine-tuned MobileNetV2 model with a multihead attention mechanism. Each panel displays both the ground truth’s bounding boxes (indicating the annotated cardiac phase intervals) and the predicted activation bounding boxes derived from Grad-CAM. The green box denotes the ground truth’s bounding box, while the black box denotes the predicted activation bounding box. Panel (**a**) shows a normal heartbeat cycle with an IoU of 0.90. Panel (**b**) depicts a heartbeat cycle with a systolic murmur and an IoU value of 0.92. Panel (**c**) presents a cycle with a diastolic murmur, yielding an IoU of 0.85, while panel (**d**) displays an abnormal cycle featuring both systolic and diastolic murmurs, with an IoU of 0.82.

Figure 13 illustrates four examples of Grad-CAM heatmaps obtained using the fine-tuned MobileNetV2 model with a multihead attention mechanism. Each panel shows both the ground truth’s bounding boxes, indicating the annotated cardiac phase intervals and the predicted activation bounding boxes derived from Grad-CAM, along with the corresponding intersection over union (IoU) values. In panel (a), a normal heartbeat cycle is shown with a Grad-CAM IoU of 0.90. Panel (b) depicts a heartbeat cycle with a systolic murmur, achieving an IoU value of 0.92. Panel (c) presents a cycle with a diastolic murmur and an IoU of 0.85, while panel (d) displays an abnormal heartbeat cycle containing both systolic and diastolic murmurs, with an IoU value of 0.82.

We follow the same procedure for the imbalanced-data experiment. Specifically, we use 10 true positive examples from the MobileNetV2 and ResNet50 models trained in the imbalanced dataset and calculate the average intersection over union (IoU) to compare the results before and after applying data balancing.

### 7.4. Experimental Setup

In our experiments, we utilized Google Colab Pro with T4 GPUs in a Python 3 environment on the Google Compute Engine’s (GCE’s) back end. We employed Librosa library for audio processing and for generating mel-spectrograms and used the LabelMe tool for annotation.

### 7.5. Evaluation Metrics

To evaluate the performance of the proposed heart-sound classification methods, we employ the following key metrics:

- **Accuracy**: Measures the overall correctness of the classification, defined as follows:
  (9) $$\mathrm{Accuracy}=\frac{\mathrm{TP}+\mathrm{TN}}{\mathrm{TP}+\mathrm{TN}+\mathrm{FP}+\mathrm{FN}}$$
  where TP, TN, FP, and FN represent the number of true positives, true negatives, false positives, and false negatives, respectively.
- **F1-Score**: A balanced measure of precision and recall, particularly useful for multiclass classifications. It is computed as follows:
  (10) $$F1-\mathrm{Score}=\frac{2\times \mathrm{Precision}\times \mathrm{Recall}}{\mathrm{Precision}+\mathrm{Recall}}$$
- **Intersection over Union (IoU) for Grad-CAM**: Measures the overlap between the model’s Grad-CAM activation map and the ground truth’s bounding box. It is defined as follows:
  (11) $$\mathrm{IoU}=\frac{\mathrm{Area}\;\mathrm{of}\;\mathrm{Overlap}}{\mathrm{Area}\;\mathrm{of}\;\mathrm{Union}}$$
  The mean IoU (mIoU) is computed as the average IoU across all the test samples, providing an interpretability measure for the model attention.

## 8. Results

**Figure 14:**
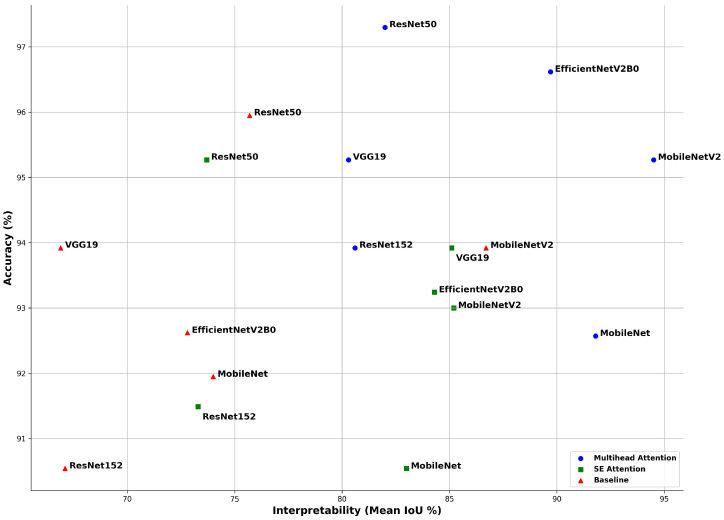
Accuracy rate and mean intersection over union (mIoU) for each model with three methods: baseline (no attention), SE attention, and multihead attention. Although the accuracy rate reflects the classification performance, the mean IoU, derived from Grad-CAM, indicates how well the model focused on relevant regions. This highlights that high accuracy rates alone do not guarantee meaningful or interpretable decisions.

In this section, we present the results of our experiments. The application of the multihead attention consistently improves the accuracy across all the models, with an average increase of 1.5–3% compared to the baseline. Figure 14 illustrates the comparative performances of six pretrained CNN models evaluated using three methods: baseline (no attention), SE attention, and multihead attention. Two core metrics were used: accuracy, which reflects the model’s classification performance, and the mean intersection over union (mIoU), which measures the alignment of the model’s predictions with diagnostically relevant regions, as identified by Grad-CAM.

The multihead attention mechanism consistently outperformed both the SE attention and baseline across most models in terms of both the accuracy and mean IoU. ResNet50 achieved the highest classification accuracy rate of 97.3%, while MobileNetV2 recorded the highest mIoU of 94.5%, indicating the strongest alignment between its predictions and critical heart-sound regions. EfficientNetV2B0 also performed exceptionally well, reaching 96.62% accuracy and a mIoU of 89.7%. MobileNet demonstrated balanced performance, with 92.57% accuracy and a 91.8% mIoU, reflecting both strong predictive capability and reliable interpretability.

The SE attention models showed a moderate improvement in interpretability compared to the baseline. For example, VGG19 and MobileNetV2 achieved mean IoU values of 85.1% and 85.2%, respectively. However, some models, such as ResNet50, underwent a drop in interpretability, despite maintaining a high accuracy rate, reporting a mean IoU of 73.7% alongside 95.27% accuracy. This suggests that SE attention, although beneficial, may not always guide the model’s focus as effectively as multihead attention.

In contrast, the baseline models trained without attention mechanisms displayed high accuracy rates but poor interpretability in many cases. For instance, VGG19 achieved an accuracy rate of 93.92%, yet its mIoU was only 66.9%, indicating that although the model produced correct predictions, it did not focus on medically meaningful areas within the signal. Similarly, ResNet152 reported 90.54% accuracy but only a 67.1% mIoU.

Overall, the results confirm that attention mechanisms not only enhance the classification accuracy but also significantly improve the model’s explainability, which is crucial for building trust in clinical applications. However, even in the best-performing multihead attention models, a substantial gap remains between the accuracy rate and mean IoU. This observation emphasizes that high classification accuracy rates alone cannot be solely be relied upon to assess a model’s reliability, especially in the context of critical decision making in healthcare, unless supported by interpretability analyses.

**Figure 15:**
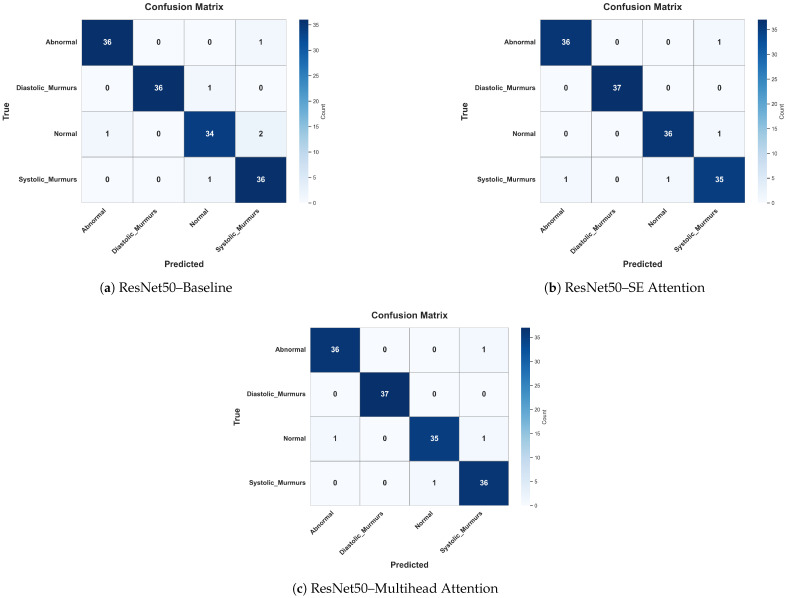
Confusion matrices for ResNet50 in three attention configurations: (**a**) baseline (no attention), (**b**) SE attention, and (**c**) multihead attention. These figures show the classification distribution across the four heart-sound classes.

To further investigate the classification behavior of ResNet50 with different attention settings, we analyzed the confusion matrices of the baseline, SE attention, and multihead attention configurations. Overall, all three settings demonstrate high true positive rates across all the classes, with multihead attention achieving the highest overall accuracy and lowest misclassification rates as shown in Figure 15.

Specifically, the multihead attention showed a perfect classification rate for diastolic murmurs and nearly perfect for abnormal, normal, and systolic murmurs, misclassifying only one instance in each. This highlights its strong generalization and localization abilities. In contrast, the SE attention model misclassified more instances, particularly, two normal cases as systolic murmurs and one diastolic murmur as normal, suggesting slightly lower reliability in distinguishing overlapping acoustic patterns. The baseline model, although strong, misclassified a systolic murmur as abnormal and a normal case as a systolic murmur, indicating that without attention, the model may struggle to focus on fine-grained features.

Importantly, these results reinforce that higher accuracy rates alone do not guarantee correct attention to clinically relevant regions, as revealed by Grad-CAM visualizations and mean IoU scores. Attention mechanisms, particularly, multihead attention, not only improved the classification performance but also led to better interpretability and class-wise clarity, which are essential for clinical trust and diagnostic adoption.

To investigate the impact of dataset balancing on the model’s interpretability, we conducted an additional experiment using MobileNetV2 (baseline)—the model that demonstrated the clearest and most consistent interpretability in the first experiment—and ResNet50, which outperformed others when combined with the multihead attention technique. This choice allowed us to reliably assess how balancing affects Grad-CAM visualizations, focusing on both the model’s explanatory quality and classification accuracy. We evaluated the model’s performances before and after applying class balancing by calculating the mean IoU and accuracy under both conditions.

**Figure 16:**
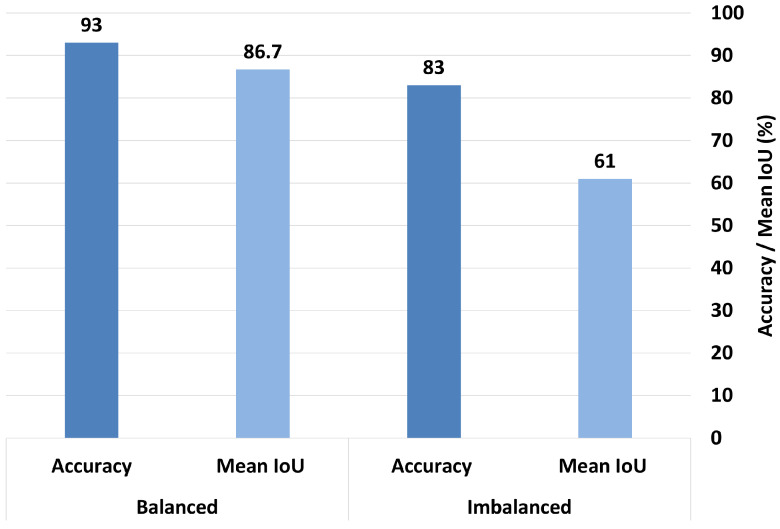
Comparison of accuracy and mean IoU for MobileNetV2 (baseline) under balanced vs. imbalanced dataset conditions.

**Figure 17:**
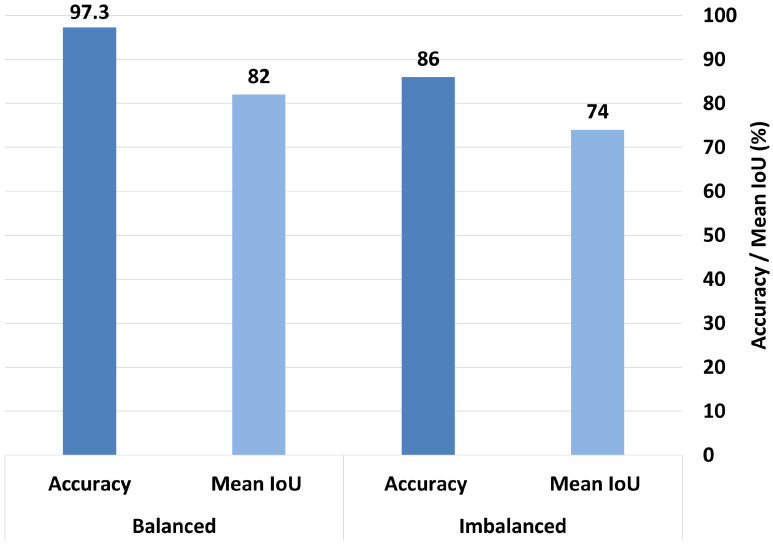
Comparison of accuracy and mean IoU for ResNet50 (multihead attention) under balanced vs. imbalanced dataset conditions.

When trained in the imbalanced dataset, MobileNetV2 achieved a mean IoU of 0.61 and an accuracy rate of 83%. After balancing the dataset, the mean IoU significantly improved to 0.867, and the accuracy increased to 93%, as shown in Figure 16. Similarly, ResNet50 trained in the imbalanced dataset achieved a mean IoU of 0.74 and an accuracy rate of 86%. After balancing, its mean IoU significantly improved to 0.82, and the accuracy rate increased substantially to 97.3%, as demonstrated in Figure 17. These findings clearly indicate that balancing the dataset plays a critical role in enhancing both the interpretability and overall classification performance of the model.

## 9. Discussion

The heart-sound classification task was conducted using six pretrained CNN models applied to a newly manually segmented version of the HeartWave dataset. To improve the performance, SE attention and multihead attention were applied. For model interpretability, Grad-CAM was used on the baseline models (without attention), the models with SE attention, and the models with multihead attention.

In general, the multihead attention method outperformed both the SE attention and baseline models in terms of both classification accuracy and interpretability. This result underscores the effectiveness of attention mechanisms, which enable models to selectively focus on the most informative regions of the input rather than treating all the data equally. Such targeted focus enhances both the predictive performance and the transparency of models’ decisions. Furthermore, the manual segmentation of the HeartWave dataset contributed significantly to the improved classification performance, achieving accuracy levels exceeding 90% even before the integration of the attention mechanisms.

However, the highest classification accuracy does not necessarily imply that a model is attending to the correct or clinically meaningful regions. For instance, although VGG19 achieved a high accuracy of 95.27% with the integration of the multihead attention mechanism, its corresponding mean intersection over union (mIoU) was 80.3%. This discrepancy indicates that strong predictive performance alone does not guarantee that the model’s decisions are based on medically relevant features. In high-risk domains, such as healthcare, it is essential that models not only deliver accurate predictions but also provide transparent and clinically interpretable justifications for their decisions in order to ensure trustworthiness and real-world applicability.

Moreover, interpretability through Grad-CAM provides insights for model optimization and further performance enhancement. By analyzing activation maps, we can identify potential areas for improvement in how the model learns and makes predictions.

Additionally, the systolic and diastolic classes include rare and complex heart conditions that are challenging to diagnose, such as pulmonic stenosis, tricuspid stenosis, and tricuspid regurgitation. This emphasizes the importance of this study, as it demonstrates how attention mechanisms can aid in the classification of difficult-to-diagnose cardiovascular conditions.

The confusion matrix comparison for ResNet50 across the three attention configurations highlights the importance of attention in improving the models’ focus and reducing misclassifications. Although the baseline model achieves a high accuracy rate of 95.95%, it shows signs of confusion between similar classes, such as normal and systolic murmurs, indicating that the model may rely on less discriminative or irrelevant features. With SE attention, the model improves in identifying harder cases, like diastolic murmurs and systolic murmurs, though some confusion between normal and murmur-related classes still persists. This suggests that SE attention helps the model to emphasize more relevant information, but does not fully resolve class overlap.

The multihead attention setup achieves the highest overall accuracy (97.3%), but its mean IoU is 82%, indicating a notable gap between the prediction confidence and precise localization or focus. This discrepancy suggests that although the model is highly accurate, it may not always base its predictions on the most relevant or interpretable features. In clinical contexts, this distinction is crucial because a model that makes correct predictions for the wrong reasons can be misleading and untrustworthy.

Therefore, although multihead attention enhances the classification performance, the difference between the accuracy and mean IoU highlights the need for interpretability alongside accuracy. Attention-based models show promise in aligning predictions with clinically meaningful features, but high accuracy rates alone do not guarantee trustworthy decision making. These results emphasize the value of incorporating explainable AI (XAI) to ensure the model’s focus aligns with clinically relevant signs, especially in medical applications.

We demonstrate that balancing the dataset is essential for achieving both improved classification performance and enhanced model interpretability. In the presence of imbalanced data, a significant gap was observed between the accuracy and mean IoU, indicating that even when the model predicted the correct class, it may not have focused on the correct regions of the image. This raises concerns about the reliability and trustworthiness of the model’s decision-making process. Conversely, after applying data balancing, this gap narrowed considerably, with the accuracy and mean IoU values becoming more aligned. This finding suggests that balanced data enable the model to make more interpretable and trustworthy predictions. Therefore, data balancing is critical not only for performance metrics but also for understanding the rationale behind the model’s outputs. In clinical applications, where explainability is a prerequisite for trust, balanced datasets are fundamental for developing credible and transparent AI-based diagnostic tools.

Although our study demonstrates promising results in classifying heart sounds and murmurs using deep-learning models with attention mechanisms, model interpretability, and manual segmentation, several limitations should be acknowledged. First, as the manual segmentation of the HeartWave dataset has been completed, increasing the number of samples could enhance the performance. Additionally, implementing cross-validation during training may further improve accuracy and model interpretability.

To support the ethical deployment of AI in clinical settings—particularly in heart-sound classification—this study emphasizes the role of explainable AI (XAI) in enhancing trust, transparency, and compliance with regulatory expectations. By applying Grad-CAM to visualize which regions of the input (e.g., S1, S2, and murmurs) contribute the most to models’ decisions, clinicians can interpret and verify the model’s behavior in light of established diagnostic knowledge [56,57]. This level of transparency helps to detect biases, ensures fairness, and reinforces the ethical responsibility of AI systems in healthcare. Moreover, explainable outputs serve as critical components in meeting the requirements of medical regulatory bodies, such as the FDA, which increasingly mandate transparency and traceability in AI-driven clinical decision support systems [58,59]. Including interpretable justifications strengthens the case for clinical adoption by enabling medical professionals to audit and trust AI predictions. Thus, the integration of XAI into our framework not only supports performance and interpretability but also contributes to the ethical, transparent, and compliant deployment of AI tools in cardiovascular diagnostics.

Future research should focus on analyzing which model layers are the most influential in the prediction process. Employing techniques like Grad-CAM can help to identify and visualize the specific layers that significantly contribute to the model’s decisions. This understanding can guide the pruning of less critical layers, leading to more efficient models without compromising the performance. Moreover, to further support the clinical meaningfulness of the results presented in this study, future work should incorporate statistical significance testing and apply inferential statistics to assess the significance of observed improvements, thereby strengthening clinical decision-making with statistically grounded evidence.

## 10. Conclusions

In this study, we proposed an explainable deep-learning framework for classifying heart sounds and murmurs, using a manually segmented dataset—HeartWave—designed to reflect clinically meaningful heartbeat cycle components: S1, systole, S2, and diastole. This structure allowed us to objectively evaluate not only the classification performance but also the model interpretability, in alignment with clinical reasoning.

To investigate the model’s focus and trustworthiness, we employed Grad-CAM as an explainable AI (XAI) technique and assessed interpretability using the mean intersection over union (mIoU) metric. We evaluated six pretrained CNN models and enhanced them with two attention mechanisms—squeeze and excitation (SE) and multihead attention. The experimental results showed that incorporating the multihead attention notably improved both the accuracy and interpretability. Specifically, ResNet50 with multihead attention achieved the highest accuracy rate of 97.3% and improved mIoU from 75.7% to 82.0%, indicating better alignment with clinically relevant regions of the signal.

These findings emphasize that high accuracy rates alone are not sufficient for reliable diagnostic support; interpretability and clinical relevance must also be considered. Our work highlights the importance of combining attention mechanisms with interpretable AI tools to build trustworthy and performance-enhanced heart-sound classification systems. To the best of our knowledge, this is the first study to use a manually segmented heart-sound dataset for objective interpretability evaluation using XAI.

## Figures and Tables

**Table 1:** Summary of related studies based on segmentation, attention mechanisms, and explainable AI (XAI) usage.

Study	Segmentation	Attention Mechanism	Explainable AI (XAI) Employed
Lilhore et al. [19], 2025			X
Li et al. [20], 2025		X	X
Han and Shaout [21], 2025	X	X	
Wu et al. [22], 2024		X	
Ranipa et al. [23], 2024		X	
Oliveira [24], 2024			X
Mohanty and Patra [25], 2024			X
Özcan [26], 2024			X
Das et al. [27], 2023		X	
Yadav et al. [28], 2023		X	
Wang et al. [29], 2023		X	
Netto et al. [30], 2023		X	
Marocchi et al. [31], 2023	X		
Bao et al. [32], 2023	X		
Cheng and Sun [33], 2023	X	X	X
Barnawi et al. [34], 2023	X		
Ren et al. [35], 2022		X	
Tian et al. [36], 2022		X	
Yin et al. [37], 2022	X	X	
Ren et al. [38], 2021		X	
Boulares et al. [7], 2021	X		
**Proposed Work**	**X**	**X**	**X**

**Table 2:** A comprehensive summary of the HeartWave dataset.

Diagnosis	Number of Recordings	Average Duration (s)
Normal	401	18.32
Aortic regurgitation	172	25.06
Aortic stenosis	104	25.69
Pulmonic stenosis	17	20.81
Pulmonary regurgitation	19	19.89
Tricuspid stenosis	18	19.95
Tricuspid regurgitation	147	23.52
Mitral stenosis	100	25.27
Mitral regurgitation	375	24.61
**Overall**	**1353**	**22.57**

**Table 3:** Distribution of heartbeat cycles in the manually segmented HeartWave dataset.

Heartbeat Class	Number of Cycles
Normal	13,905
Systolic Murmurs	10,849
Diastolic Murmurs	1625
Systolic and Diastolic Murmurs	4183
**Total**	**30,562**

**Table 4:** Distribution of samples in the HeartWave dataset used in our experiments.

Class	Balanced Dataset	Imbalanced Dataset
Normal	237	2000
Systolic Murmurs	237	1500
Diastolic Murmurs	237	237
Abnormal (Systolic + Diastolic)	237	1000

**Table 5:** Summary of selected pretrained CNN models used in this study [55].

Model	Depth	Size (MB)	Parameters
ResNet50	107	98	25.6 M
ResNet152	311	232	60.4 M
MobileNet	55	16	4.3 M
MobileNetV2	105	14	3.5 M
VGG19	19	549	143.7 M
EfficientNetV2B0	273	29	7.2 M

**Table 6:** Hyperparameters for fine-tuning the pretrained CNN models.

Parameter	Value
Dataset Split	70% Training, 15% Validation, 15% Testing
Input Image Size	224 × 224 pixels
Batch Size	5
Learning Rate	0.0001
Optimizer	Adam
Early Stopping	Monitor: Validation Categorical Accuracy, Patience: 10 epochs, Restore Best Weights

## Data Availability

The original contributions presented in the study are included in the article, further inquiries can be directed to the corresponding author.
